# “Bath Salts” Intoxication: A Case Report

**DOI:** 10.1155/2012/976314

**Published:** 2012-03-25

**Authors:** Michael Falgiani, Bobby Desai, Matt Ryan

**Affiliations:** Department of Emergency Medicine, University of Florida College of Medicine, 1329 SW 16th Street, P.O. BOX 100186, Gainesville, FL 32610-0186, USA

## Abstract

We present a case of a potentially lethal ingestion of “Bath Salts.” After presentation, we briefly review the epidemiology and pathology of “bath salts” ingestion.

## 1. Background

“Bath salts” is a new substance, initially legal in all states, that has become an ever increasing problem with intoxication in young adults. This case presents an example of a life-threatening ingestion of “bath salts” and a review of the pathophysiology of bath salts as well as some epidemiology.

## 2. Case Presentation

We present a case of a 29-year-old female brought in to the Emergency Department (ED) by ambulance for altered mental status. She had a friend who arrived with her who provided the majority of the history. Per the friend, both girls went to a rave party 2 nights prior and were snorting bath salts, among other drugs. They continued using bath salts for the following two days. The friend called 911 when she found our patient curled up in a corner of the friend's home. Paramedics found our patient with a Glasgow Coma Score (GCS) of 11, on the floor. The patient was noted to have in her possession two prescription bottles labeled trazodone and lithium, with three different types of pills found in those two bottles. Based on the patients' condition upon arrival no further history was available to be obtained at that time.

On arrival the patient's vital signs were pulse of 85, respiratory rate of 17, blood pressure of 114/79, and an O2 saturation of 100% on room air. Paramedics obtained a finger stick glucose which measured 106. The patient had another finger stick glucose upon arrival to the ED which was 71. On physical exam, the patient appeared obtunded. Her head, ears, eyes, neck, and throat (HEENT) exam revealed 1 mm pupils bilaterally that were sluggishly reactive and equal bilaterally. The rest of her HEENT exam was unremarkable. Cardiovascular exam showed a regular rate and rhythm with strong pulses throughout. Her lungs were clear to auscultation bilaterally. Her abdomen was soft, nontender, and nondistended. On neurological exam, pt was noted to be a GCS of 11 (3 points for eyes, 6 points for motor and 2 points for verbal).

The patient had blood sent to the lab for the following studies. Her complete blood count (CBC) revealed a white blood cell count of 9.6, hemoglobin of 12.5, hematocrit of 40.5, and a platelet count of 240. Her INR was 1.0. We obtained cardiac markers including creatine kinase and troponin, both of which were unremarkable. Her basic metabolic panel (BMP) was within normal limits with an anion gap of 8. Her alcohol level was zero. Her urine pregnancy test was negative, and her urinalysis was unremarkable. Her urine drug screen was positive for both benzodiazepines and cocaine. Her lithium level was noted to be 2.5. We obtained an electrocardiogram (EKG) which showed a normal sinus rhythm with a rate of 75, a normal axis, normal intervals, and no ST/T abnormalities noted. Her chest X-ray was notable for questionable absence of lung markings in the right apex.

Her management in the emergency department began with initial stabilization. She was given a 1-liter bolus of normal saline (NS) intravenously (IV). She was administered 0.4 mg of Narcan IV, for which there was little response. At that point, poison control was contacted. The patient remained in stable condition and was admitted to the Internal Medicine service for further evaluation and observation. We later found out this patient had a history of bipolar disorder and polysubstance abuse. She was currently prescribed lithium and trazodone. When pt became more responsive, she admitted to a 2-day binge of drug use including snorting bath salts, followed by the use of valium, lithium, and trazodone to help her sleep. The patient was observed on the Internal Medicine service until she returned to baseline. Her lithium level was tracked and trended downward to 0.6 upon her discharge. This patient was discharged to home with close psychiatry followup.

## 3. Discussion

Bath salts are becoming increasingly popular as a form of recreational drug use in our country. These “bath salts” are being used as an alternative to street drugs such as cocaine. These bath salts have no use for bathing, although sold under the name “bath salts,” a term usually meant to describe a type of powdered soap to be added to bath water for cleaning. Some of the more common street names for bath salts include red dove, vanilla sky, ivory wave, bliss, white lightning, super coke, tranquility, zoom, and magic. The chemical compounds that cause the symptoms associated with bath salts are mephedrone and methylenedioxypyrovalerone (MDPV). The parent drug is cathinone from the plant *Catha edulis *[1]. The cost for 300 milligrams (mg) is approx. $20. It is typically sold as a white powder or in crystal form, and the usual dose is between 50 mg and 300 mg. The substance can be snorted as a powder or smoked as a crystal form. It can also be injected. This substance can be purchased over the internet or locally in “head shops” or certain convenience stores. Many of these internet sites are now international, and as such difficult to regulate. Bath salts work by stimulating release and inhibiting the reuptake of norepinephrine, serotonin, and dopamine. Bath Salts cause symptoms consistent with a sympathomimetic toxodrome [2]. The most common symptoms include hallucinations, paranoia, insomnia, agitation, and suicidal thoughts. These symptoms can mimic acute psychosis [3]. This chemical can also cause rapid heart rates, palpitations, chest pain, headaches, and seizures. There have been reports of patients developing myocarditis and methemoglobinemia after snorting large dosages of bath salts, up to 1 gram. There has also been at least one reported death in Michigan due to bath salts [4] as well as at least 5 deaths in Europe [2]. The mainstay of therapy in the ED is supportive treatment along with the use of benzodiazepines as needed for agitation and seizures.

This is becoming a nationwide problem. Poison control has noted calls from at least 25 states. Louisiana had a significant problem with bath salts in 2010. They had more than 125 calls to poison control from October to December 2010. The state enacted an emergency order to outlaw bath salts. As of June, over 3,400 calls have been received by poison control centers across the country this year, in comparison, there were only 303 cases reported in 2010 [5]. The drug enforcement administration (DEA) has taken action as of the 7th September. They have invoked an emergency scheduling authority to control the substances found in bath salts. This action will make it illegal to sell or posses these chemicals for a one-year period as they further study these chemicals [6].

## Abbreviations

ED: Emergency Department
GCS: Glasgow Coma Score
HEENT: Head, ears, eyes, neck, and throat
CBC: Complete blood count
BMP: Basic metabolic panel
EKG: Electrocardiogram
NS: Normal saline
IV: Intravenously
mg: Milligrams
MDPV: Methylenedioxypyrovalerone
DEA: Drug enforcement administration.
